# Aerobic capacity and skeletal muscle characteristics in glycogen storage disease IIIa: an observational study

**DOI:** 10.1186/s13023-022-02184-1

**Published:** 2022-01-31

**Authors:** Philip J. Hennis, Elaine Murphy, Rick I. Meijer, Robin H. Lachmann, Radha Ramachandran, Claire Bordoli, Gurinder Rayat, David J. Tomlinson

**Affiliations:** 1 Sport, Health and Performance Enhancement (SHAPE) Research Centre, Nottingham Trent University, Clifton Lane, Clifton, Nottingham, NG11 8NS UK; 2 Charles Dent Metabolic Unit, The National Hospital for Neurology and Neurosurgery, London, UK; 3 Section of Vascular Medicine, Department of Internal Medicine, Amsterdam UMC, Amsterdam, The Netherlands; 4 Department of Adult Inherited Metabolic Disease, St Thomas’ Hospital, London, UK; 5 Shackleton Department of Anaesthetics, Perioperative Medicine Team, University Hospital Southampton NHS Foundation Trust, Tremona Road, Southampton, SO16 6YD UK; 6 Musculoskeletal Science and Sports Medicine Research Centre, Manchester Metropolitan University, Manchester, UK

**Keywords:** Skeletal muscle, Glycogen storage disease type IIIa, Maximum voluntary contraction, Aerobic capacity

## Abstract

**Background:**

Individuals with glycogen storage disease IIIa (GSD IIIa) (OMIM #232400) experience muscle weakness and exercise limitation that worsen through adulthood. However, normative data for markers of physical capacity, such as strength and cardiovascular fitness, are limited. Furthermore, the impact of the disease on muscle size and quality is unstudied in weight bearing skeletal muscle, a key predictor of physical function. We aim to produce normative reference values of aerobic capacity and strength in individuals with GSD IIIa, and to investigate the role of muscle size and quality on exercise impairment.

**Results:**

Peak oxygen uptake (V̇O_2_peak) was lower in the individuals with GSD IIIa than predicted based on demographic data (17.0 (9.0) ml/kg/min, 53 (24)% of predicted, *p* = 0.001). Knee extension maximum voluntary contraction (MVC) was also substantially lower than age matched predicted values (MVC: 146 (116) Nm, 57% predicted, *p* = 0.045), though no difference was found in MVC relative to body mass (1.88 (2.74) Nm/kg, 61% of predicted, *p* = 0.263). There was a strong association between aerobic capacity and maximal leg strength (r = 0.920; *p* = 0.003). Substantial inter-individual variation was present, with a high physical capacity group that had normal leg strength (MVC), and relatively high V̇O_2_peak, and a low physical capacity that display impaired strength and substantially lower V̇O_2_peak. The higher physical capacity sub-group were younger, had larger Vastus Lateralis (VL) muscles, greater muscle quality, undertook more physical activity (PA), and reported higher health-related quality of life.

**Conclusions:**

V̇O_2_peak and knee extension strength are lower in individuals with GSD IIIa than predicted based on their demographic data. Patients with higher physical capacity have superior muscle size and structure characteristics and higher health-related quality of life, than those with lower physical capacity. This study provides normative values of these important markers of physical capacity.

**Supplementary Information:**

The online version contains supplementary material available at 10.1186/s13023-022-02184-1.

## Background

Glycogen storage disease IIIa (GSD IIIa) (OMIM #232400) is a rare inherited metabolic disorder caused by pathogenic variants in the *AGL* gene which spans 85 kb of DNA on chromosome 1p21.2 and is composed of 35 exons [1]. Pathogenic mutations of the *AGL* gene result in glycogen debrancher enzyme deficiency (GDE) which impedes glycogenolysis and results in excessive glycogen storage. The debranching enzyme is a single polypeptide with two catalytic sites, amylo-1,6-glucosidase (EC 3.2.1.33) and 4-alpha-glucanotransferase (EC 2.4.1.25). To date at least 110 disease causing variants have been reported, illustrating a high degree of genetic heterogeneity [2]. GSD IIIa primarily affects the liver, skeletal muscle and the heart, causing hypoglycaemia, hepatomegaly and (cardio)myopathy [3, 4]. Because of these pathologies, patients suffer from muscle weakness and exercise limitation that worsen through adulthood [3, 5] and can result in patients becoming wheelchair bound [6].

Experts recognise that exercise is likely to be a useful tool for the assessment of functional status and as a treatment strategy to combat some pathophysiological consequences of GSD IIIa, such as myopathy, low bone mineral density and hypoglycaemia [7]. However, they also state that they are unable to provide strong guidance related to the prescription of regular exercise for patients due to a lack of information [7]. This lack of information and thus guidance creates difficulties for physicians considering to use exercise testing/training in their clinical practice. One such omission in the research literature is the lack of sufficient normative reference data for GSD IIIa to aid interpretation of cardiovascular fitness and muscular strength outcomes. At present, normative data for cardiovascular fitness is limited to 12 individuals [8–10] whilst leg strength has been reported in 18 patients [11]. The current study adds normative data on skeletal muscle size and quality. We further employed commonly used technologies and techniques to add to the small literature base providing normative reference values for cardiovascular fitness and quadriceps strength, and studied whether these parameters are associated.

Skeletal muscle size and quality are important markers of physical health, predicting physical performance [12], gait variability [13], and fall risk [14]. In other diseases characterised by myopathy, such as muscular dystrophy, muscle size and quality are lower than predicted for their age and sex, and account for differences in physical capacity [15]. As yet, the relationships between markers of physical capacity and muscle structure are largely unstudied in GSD IIIa and warrant investigation to help further understand the relative importance of metabolic, muscular, neuromuscular, and cardiorespiratory function on physical capacity in these patients. Furthermore, in other populations, physical activity (PA) and sedentary behaviour (SB) are strongly associated with exercise capacity and muscle strength [16, 17], but their associations in GSD IIIa are unstudied. As such, we examined the association between these movement behaviours and aerobic fitness and leg strength.

### Purpose

To produce normative reference values of aerobic capacity and strength in individuals with GSD III and to investigate the role of muscle size and quality on exercise impairment.

## Results

The demographic and clinical characteristics of the seven participants are shown in Table 1. Briefly, three were female, and the mean (standard deviation) age and height of participants were 37 (11) years and 179 (10) cm, respectively. Median (IQ range) for body mass was 80.6 (8.0) kg. Disease severity (or impact on daily life) varied across the cohort. Participants 1 and 6 had hepatomegaly. Seven participants also had splenomegaly, without imaging evidence of portal hypertension. Ejection fraction (transthoracic echocardiogram) was normal in all patients (range 57–76%). Left ventricle maximum wall thickness (LV MWT) > 1.1 cm, found in two patients, was considered indicative of left ventricular hypertrophy (Table 1). All participants had elevated resting creatine kinase (CK) activity. Three individuals did not take any medication, whilst four took one or more of the following; Allopurinol, Vitamin D, Bisphosphonate (Table 1).

**Table 1:** Participant characteristics, ordered by V̇O_2_peak (from highest to lowest)

Participant	Patient demographics				Clinical Characteristics				Dietary information and medications			
	Gender	Age (years)	Height (cm)	Body Mass (kg)	Cardiac hypertrophy (Y/N)	hepatomegaly (Y/N)	Creatine kinase (RR: 26–140 IU/L)	Triglycerides (mmol/L)	Higher protein diet	Protein supplement	UCCS	Medication
1	M	27	181.0	81.4	Y*	Y	6592	1.2	Y	N	Y	A
2	F	28	173.1	73.3	N	Y	641	1.1	N	N	N	None
3	M	26	193.2	80.6	N	Y	3842	1.6	Y	Y	N	A, D
4	M	48	180.3	69.5	N	Y	1756	2.2	Y	Y (Int)	N	B, D
5	F	50	173.9	81.1	N	Y	1266	1.4	Y	N	N	None
6	F	45	162.3	78.9	Y^#^	N	2442	1.1	N	N	N	None
7	M	32	188.0	112	N	Y	2682	1.5	Y	Y	Y	D
Mean (SD)	–	37 (11)	179 (10)		–	–	2746 (1987)	1.4 (0.4)	–	–	–	–
Median (IQ range)	–			80.6 (8.0)	–	–		–	–	–	–	–

Int, Intermittent; Cardiac hypertrophy: *Y1, Maximal wall thickness 14 mm (asymmetric septal), ^#^Y6, Maximal wall thickness 13 mm (basal septal); UCCS: uncooked cornstarch: Y, 50 g nightly; A, Allopurinol; D, Vitamin D; B, Bisphosphonate

A formal diet diary was not completed as part of this study. Participants were advised to continue with their normal diet and no specific recommendations were made with regard to dietary intake immediately before testing. Whilst our general clinical recommendation is that individuals with GSD IIIa consume a diet higher in protein, with a preference for complex carbohydrates rather than simple sugars [7, 18], in practice review of clinical records indicated that the participants’ diets did not all follow this approach (Table 1). Five participants consumed a higher protein diet, three with the addition of protein supplementation (these three participants were aiming to consume 2 g/kg/day protein). Two participants included regular additional uncooked cornstarch (UCCS) in their diet (both took 50 g UCCS before bed).

### Cardio-respiratory

Peak oxygen uptake (V̇O_2_peak) was lower in participants than predicted based on their demographic data (17.0 (9.0) ml/kg/min, 53 (24)% of predicted, *p* = 0.001), as was peak work rate (Median: 54 (IQ range: 118)) watts, 30 (38)% predicted, *p* = 0.018), and peak heart rate (143 (28) bpm, 78 (13)% predicted *p* = 0.005) (Table 2). Peak minute ventilation (V̇E) was 37 (20) L/min equivalent to 22 (8)% of predicted maximum voluntary ventilation. Anaerobic threshold (AT) was only identifiable in 2 participants. These were 15 and 16 ml/kg/min for participants 1 and 2, respectively. The AT could not be determined in the other participants due to not being reached (participants 4, 5, 6 and 7) and atypical expiratory gas exchanges patterns prohibiting AT determination (participant 3).

**Table 2:** Cardio-respiratory properties, ordered by V̇O_2_ peak

Participant							Cardio-respiratory characteristics						PA characteristics (min/day)			
	Ramp duration (min:s)	V̇O_2_peak (ml/kg/min)			V̇O_2_peak (% of predicted)	Peak WR (W)	Peak WR (% of Predicted)	Peak V̇E (L/min)	V̇E (% of Predicted)	Peak Heart Rate (BPM)	Heart rate (% of Predicted)	Peak RER	SB	LIPA	MPA	VPA
1	10:40	27.1			67	159	63	53	29	179	93	0.97	410	313	227	0
2	09:49	25.9			91	146	82	56	33	169	88	0.99	650	170	129	11
3	10:16	25.7			63	153	52	63	27	163	84	0.91	637	200	124	0
4	03:39	15.0			46	54	25	27	18	130	76	0.88	669	218	73	0
5	05:36	9.4			45	41	28	16	16	139	82	0.81	448	459	53	0
6	03:43	8.7			37	35	30	21	21	121	69	0.93	640	163	157	0
7	02:32	7.0			22	25	9	22	11	102	54	0.85	678	228	34	0
Mean (SD)	06:36 (03:32)	17.0 (9.0)			53 (24)			37 (20)	22 (8)	143 (28)	78 (13)	0.91 (0.06)	590 (112)	250 (105)	114 (67)	1.6 (0)
Median (IQ range)						54 (118)	30 (38)						640 (221)			0 (0)

V̇O_2_, oxygen utilisation; WR, work rate, V̇E, minute ventilation; RER, respiratory exchange ratio; PA, physical activity; SB, sedentary behaviour; LIPA, Light-intensity physical activity; MPA, moderate-intensity physical activity; HPA, High-intensity physical activity

### Muscle strength and size

Absolute maximum voluntary contraction (MVC) was markedly lower in participants than predicted (*p* = 0.045) (Table 3). A noticeable difference was also present between MVC relative to body mass and age matched predicted values, however, no significant differences were observed (*p* = 0.176). Rate of torque development (RTD) could only be determined in 5 individuals and the median (IQ range) was 328 (767) Nm s. Mean muscle volume, the physiological cross sectional area (PCSA) and muscle quality were 516 (240) cm^3^, 77 (40) cm^2^, and 1.60 (0.70) Nm cm^2^, respectively (Table 3).

**Table 3:** Descriptive skeletal muscle structural and functional characteristics, ordered by V̇O_2_peak (from highest to lowest)

Participant	Functional characteristics							Structural characteristics		
	MVC (Nm)	Predicted MVC (Nm)	Percentage achieved (%)	MVC/BM (Nm/kg)	Predicted MVC/BM (Nm/kg)	Percentage achieved (%)	RTD 0–200 ms (Nm∙s)	Vastus Lateralis Muscle Volume (cm^3^)	Vastus Lateralis PCSA (cm^2^)	Muscle Quality (Nm∙cm^2^)
1	267	281	95	3.28	2.67	123	975	726	126	2.13
2	225	210	107	3.07	2.87	107	–	544	81	2.77
3	309	302	102	3.83	3.75	102	965	940	139	2.23
4	47	226	21	0.68	3.25	21	214	273	36	1.32
5	56	198	28	0.64	2.44	26	–	323	48	1.17
6	43	174	25	0.54	2.21	24	192	393	54	0.79
7	77	339	23	0.69	3.03	23	328	419	54	1.44
Mean (SD)	146 (116)	247 (61)	57 (42)		2.89 (0.52)	61 (47)		516 (240)	77 (40)	1.60 (0.70)
Median (IQ range)			28 (80)	1.82 (2.64)		26 (84)	328 (767)			

*MVC* maximum voluntary contraction, *BM* body mass, *RTD* rate of torque development, *PCSA* physiological cross-sectional area

### Associations between cardio-respiratory fitness, muscle strength, and muscle characteristics

There was a strong association between aerobic capacity and maximal leg strength. Pearson’s correlations identified a significant positive association between V̇O_2_peak and absolute MVC (r = 0.920; *p* = 0.003). Spearman’s correlations revealed no association between V̇O_2_peak and MVC relative to body mass (r = 0.679; *p* = 0.094), or between V̇O_2_peak and RTD (r = 0.700; *p* = 0.188). Aerobic capacity was also associated with muscle structural characteristics. Pearson’s correlations identified significant positive associations between V̇O_2_peak and Vastus Lateralis (VL) muscle volume (r = 0.771; *p* = 0.043), PCSA (r = 0.819; *p* = 0.024), and muscle quality (r = 0.884; *p* = 0.008). Pearson correlations revealed VL muscle volume (r = 0.943; *p* = 0.001), PCSA (r = 0.957; *p* = 0.001) and muscle quality (r = 0.863; *p* = 0.012) were all positively correlated with knee extension MVC.

### Physical activity, quality of life and pain

Participants’ PA levels are reported in Table 2. Analysis revealed that only two (participants 1 and 5) were classified as ambulatory (< 8 h (480 min) of SB) and the remaining as sedentary. Light-intensity physical activity (LIPA) and moderate-intensity physical activity (MPA) accounted for the majority of movement, 60% and 28%, respectively. High-intensity physical activity (HPA) was not achieved by six of the seven participants, and the remaining participant recorded an average of 11 min of HPA per day.

Self-reported health status results taken from the SF-36 are reported in Additional file 1: Table S1. All physical and emotional outcomes were lower in participants than would be expected for their age and gender [19]. Mental health variables were less affected, with emotional well-being and role limitations due to emotional problems with median values of 100% and 65% of their predicted values, respectively. In contrast, variables related to physical health were much lower than predicted (median physical functioning, 41% predicted; role limitations due to physical health, 0% predicted; energy/fatigue, 29% predicted).

Current pain intensity at baseline (taken pre-exercise) was 1.6 (1.8), and did not alter when assessed the day after the exercise trials (1.6 (1.6), *p* = 1.000). Maximum pain endured over the past 24 h was also no different the day following exercise when compared to baseline, 2.9 ± 2.1 and 3.0 ± 2.8 (*p* = 0.846), respectively.

### Higher physical capacity vs lower physical capacity

In comparison to predicted normative values, participants had substantially lower aerobic fitness (V̇O_2_peak) and muscle strength (MVC). However, within the group, substantial inter-individual variation was present across these variables, such that two separate groups of patients emerged. A higher physical capacity group, comprised of participants 1, 2, 3, that maintained normal maximal leg strength (MVC: 101% of predicted) and relatively high aerobic capacity (V̇O_2_peak: 73% of predicted), and a lower physical capacity group, comprised of participants 4, 5, 6, 7 who achieved 24% and 38% of their predicted values for MVC and V̇O_2_peak, respectively. The sub-group with greater physical capacities had greater muscle volume (737 vs. 352cm^3^), PCSA (115 vs. 48cm^2^) and muscle quality (2.38 vs. 1.18 Nm∙cm^2^). They were younger (27 ± 1 yrs) than those with less strength (44 ± 8 yrs), tended to self-report higher physical functioning (Additional file 1), and spent more time undertaking moderate to vigorous physical activity (MVPA) (Table 2).

## Discussion

This study demonstrates that V̇O_2_peak and knee extension strength are lower in individuals with GSD IIIa than predicted based on their demographic data. Muscle size and quality were positively associated with both V̇O_2_peak and MVC, implicating muscle atrophy and neuromuscular impairment for the functional decline observed in this cohort. Interestingly, a high physical capacity group emerged that had normal leg strength (MVC) and relatively high V̇O_2_peak, and a low physical capacity that display impaired strength and substantially lower V̇O_2_peak. The higher physical capacity sub-group were younger, had superior muscle size and quality, and tended to undertake more PA and report higher health-related quality of life. This study demonstrates that Peak V̇O_2_ and isometric maximal strength measurements can be undertaken safely in this population and provides normative values of these important markers of physical capacity.

Peak V̇O_2_ in this cohort was 17.0 (9.0) ml/kg/min, which is somewhat lower than previously reported in six patients with GSD IIIa (25.4 ± 5.1 mL/kg/min) [9]. The difference between studies is removed when patients are compared to control/normative values, with the current sample achieving 54% of predicted based on the demographic information and the sample collected by Preisler et al. [9] achieving 55% that of their matched control. This indicates that the difference in V̇O_2_peak between studies is likely due to the current study recruiting older individuals (37 (11) vs 27 (8) years) with higher disease severity. The deficit in V̇O_2_peak was different amongst the group, with participants achieving between 22 and 91% of their predicted value. Hoogeveen et al. [10] also identified a large range in V̇O_2_peak but with values generally higher than the current study (range: 46–105% of predicted). A key factor explaining this variation in the current study appears to be age/disease progression, with those achieving a higher V̇O_2_peak tending to be younger. However, in contrast, Hoogeveen et al. [10] found older participants had the highest V̇O_2_peak relative to predicted. This discrepancy could be attributable to differences in the genetic and environmental characteristics of the two samples.

The cause of lower V̇O_2_peak in GSD IIIa will reflect the underlying pathophysiology of the disease, and these are likely different from those in healthy people. In healthy individuals, the attainment of V̇O_2_peak is typically attributed to cardiovascular limitation [20] with heart rate reaching maximal levels when exercise ceases. In the current study, no participants with GSD IIIa reached their predicted maximum heart rate, indicating that factors other than cardiovascular limitations were responsible for exercise cessation. Furthermore, the associations between V̇O_2_peak, MVC and muscle size provides evidence of the role muscle weakness has on limiting aerobic capacity, whilst the low peak respiratory exchange ratio (RER) supports previous studies demonstrating impaired skeletal muscle glycogenosis impedes exercise performance [8, 9].

The sensitivity of V̇O_2_peak to impairments in cardiovascular, respiratory, metabolic and neuromuscular function make it an ideal tool for the assessment of physical function in diseases with complex pathology, such as GSD IIIa. In other forms of GSD, cardiopulmonary exercise testing (CPET) has been successfully used to assess the effectiveness of various therapeutic interventions, with improvements in V̇O_2_peak noted following enzyme replacement therapy (ERT) in GSD II [21] and exercise training in GSDs II and V [22]. Now that new therapies are in development for GSD IIIa [23, 24], and exercise training is increasingly recognised as an adjunct therapy [7], future studies should consider measuring V̇O_2_ peak to evaluate the efficacy of these interventions.

Overall, study participants achieved 57% of their predicted MVC. However, results indicate that 3 of the study participants (participants 1–3) would be classed as having a normal maximal strength capacity compared with their predicted age and sex matched counterparts (101% of predicted) [25], whilst 4 people (participants 5–8) had impaired knee extensor strength (24% of predicted). This phenotypic variation in leg strength was also noted by Decoste et al. [11], whose patients’ strength ranged from approximately 5 to 80% of predicted. In the current study, those with higher strength tended to be younger (27 ± 1 years) than those with less strength (44 ± 8 years) indicating disease progression is a primary cause. These findings of a reduction in strength with age would be consistent with the cross sectional study by Decostre et al. [11] which noted a drop in muscle function around the 3rd decade of life at a rate of 0.7% per year. Knee extensor strength is an important health marker as it contributes to habitual functional activities such as gait speed [26], rising from a chair [27], and stair negotiation [28]. As such, individuals with GSD IIIa may benefit from interventions designed to increase/maintain leg strength, such as resistance training, which have been successful with other low strength cohorts such as in adults with Pompe [29] and limb-girdle, Becker, and facioscapulohumeral dystrophies [30].

This is the first study to examine the link between muscle size and MVC capacity in individuals with GSD IIIa and interestingly both variables were shown to positively correlate with each other, in line with previous literature that isometric MVC is proportional to the PCSA [31]. This suggests that smaller muscle size is partially responsible for the lower maximal strength alongside potential neuromuscular deficiencies in agonist activation noted during healthy ageing [32] and neuropathy previously observed in GSD IIIa [6], however these hypotheses need to be confirmed in future longitudinal investigations.

Muscle quality (MQ) is the ability to produce force relative to contractile tissue mass/volume and is a key determinant of physical function and mobility in later life [33]. Interestingly, muscle quality was lower in participants when compared against age and sex matched normative data [34]. These findings support a decrease in the intrinsic fibre properties especially as individuals with GSD IIIa age, and this explanation is additionally supported by a greater decrement in RTD. Potential mechanisms to explain this decrement include neuromuscular and myopathic manifestations in skeletal muscle which become exacerbated with age [6]. The functional consequence for a reduction in muscle quality could lead to functional impairment [12], gait variability [13], and fall risk [14].

The participants with GSD IIIa had low PA levels with the majority of the study sample classed as sedentary (> 8 h of SB) [35, 36]. Self-reported health status of our cohort was also lower than the general population [19]. The GSD IIIa group with higher physical capacity, those with normal leg strength and relatively high aerobic capacity, undertook noticeably more MVPA (164 (55) min/day) than those with lower physical capacity (79 (54) min/day). They also tended to self-report higher levels of physical health, though levels still tended to be lower than age and sex matched normative values. These results indicate that individuals with GSD IIIa with high physical capacity undertake more PA and enjoy better health, or conversely, it indicates that high PA and better health status led to greater physical capacity in these patients. Using the current cross-sectional study design it is impossible to know the direction of this association, though both these scenarios are likely true to some extent. Longitudinal assessment of changes in movement behaviour, physical capacity and health will help us more clearly understand the interplay between these important behaviours and health outcomes, whilst also informing potential exercise interventions.

### Implications

Expert guidelines highlight the need for regular assessments of strength and aerobic capacity in individuals with GSD IIIa to monitor status and guide exercise training [7]. However, to fully interpret any exercise results, clinicians require normative data in the populations of interest. This study shows that aerobic capacity and maximal strength are plausible outcome markers of physical function in GSD IIIa and may be useful given exercise intolerance is a major complication associated with the disease.

### Strengths and limitations

The current study employed a relatively small sample size (n = 7) which reflects the rare nature of the disease and the difficulties this creates for recruitment. Small sample sizes limit the utility of data for the provision of normative physical capacity values in rare disorders. To combat this, our tests were conducted using standard techniques and equipment that are available in many hospitals and universities, and we presented the results at an individual level. Our hope is that future studies will use similar testing techniques to those used here and combine our data with theirs to create a larger, more representative, data set.

A strength of the current study is that we were able to demonstrate that assessment of aerobic capacity and leg strength was achievable in individuals with GSD IIIa. However, our first participant reported suffering from leg pain and contractures following the test, which resulted in an elevation in CK concentration that resolved after 1 week. The patient felt that these symptoms were brought on by the strength tests, which at the time consisted of three knee extension isometric contractions at 70°, 80° and 90°, and two isokinetic knee extension and flexion at 60°/sec and 120°/sec (both concentric contractions) with 90–120 s rest between contractions. Muscle pain was noted following isokinetic contractions in the participant and may have implications for developing future training protocols (low %1RM and low volume). However, in response to this incident we extended the rest time between contractions to 5–10 min and excluded isokinetic contractions from the trial and no further patient reported any adverse side effects.

The current study compared the physiological responses of participants with GSD IIIa with predicted values, rather than using a control group. Though comparing patients’ results to normative data is not optimal, selecting appropriate controls to compare with this small sample of individuals with GSD IIIa would have been problematic and open to selection bias. We concluded that the benefit of comparing our results with data collected on hundreds of healthy controls and being able to adjust for age, height, weight and sex warranted the comparison to normative values in our study.

The measurement of V̇O_2_peak is effort dependent so it is difficult to know with certainty if the values we obtained actually represent the participants’ aerobic capacity. To verify V̇O_2_peak, the participant can perform a second, constant work rate test at a severe intensity to determine if an equivalent V̇O_2_ is achieved [37]. We did not employ this additional severe-intensity phase as we believed it may encourage muscle damage and contractures in our cohort. However, we believe our cohort was highly motivated to exercise and that the measure of V̇O_2_peak is reflective of their exercise capacity.

Finally, activity monitoring was performed in the week following the exercise trials. It is possible that completing the exercise tests had residual fatiguing effects and lead us to underestimate PA. Future studies should avoid this oversight by monitoring PA at a different time or by allowing a recovery period prior to starting PA monitoring.

## Conclusion

V̇O_2_peak and MVC are lower in individuals with GSD IIIa than would be expected. The data is intended to provide some normative reference data of cardiovascular fitness and muscular strength outcomes in GSD IIIa. The mechanisms responsible for the impairment in V̇O_2_peak in GSD IIIa are yet to be fully determined, but the associations between V̇O_2_peak, MVC, muscle size and quality highlight the role of muscle weakness. The deficit in physical capacity was highly variable in the cohort and though older age accounts for some of the decline more research is needed to understand the impact of lifestyle/treatment choices, such as diet and PA on exercise capacity.

## Methods

### Patients

In this descriptive study, participants were recruited from the Charles Dent Metabolic Unit, National Hospital for Neurology and Neurosurgery, London and the Department of Adult Inherited Metabolic Disease, St Thomas’ Hospital, London. Adult patients (> = 18 years) with a diagnosis of GSD IIIa (confirmed by reduced GDE enzyme activity and/or *AGL* genetic analysis) were eligible for inclusion. Participants were excluded from participating if they were pregnant, had absolute contraindications to exercise testing, as advised by the American Thoracic Society/American College of Chest Physicians statement on cardiopulmonary exercise testing [38], or if they were deemed unable to safely mount/dismount an exercise bike. Following implementation of these criteria, 7 individuals (3 female) provided informed consent to participate in the study. Five of the participants walked independently, one required the use of a walking aid, and one required a wheelchair to travel over longer distances. The study was approved by the South Central—Berkshire B Research Ethics Committee (16/SC/0663).

### Protocols

Following informed consent, on the day of testing participants had baseline (non-fasting) blood tests taken (CK, lipid profile, glucose, urate). All exercise tests were conducted in an exercise physiology laboratory in the presence of a medical doctor and an exercise physiologist. Tests were conducted in a specific order to reduce the likelihood of one test affecting another, and to allow adequate time for recovery between exercise bouts. First, resting measures of body composition and pain were taken. Patients then undertook the two exercise bouts. They first completed a CPET and then, following a minimum of two hours rest, during which patients ate lunch and completed questionnaires (described below), they undertook knee extension exercise to determine MVC. Patients were not restricted from eating or drinking for the duration of the study. An isotonic sports drink providing 32.5 g carbohydrates (18.0 g sugar) was made available for each participant and, where diet allowed, patients were encouraged to drink it prior to the exercise tests to potentially reduce exercise-induced muscle pain [8]. Throughout the day, patients were asked to stop exercise if they believed continuing might result in muscle soreness and damage.

### Body composition

On arrival to the laboratory, patients had their height (Seca 217 stadiometer, Seca, Hamburg, Germany), weight (Seca 761 scales, Seca, Hamburg, Germany), and body composition measured using bioelectrical impedance (MC-980MA PLUS, Tanita Corporation, Tokyo, Japan).

### Cardio-pulmonary exercise testing

A symptom-limited, incremental ramp cycling protocol to volitional exhaustion was performed to determine V̇O_2_peak and AT using breath-by-breath gas analysis (Vyntus CPX Metabolic Cart, CareFusion, Höchberg, Germany). The test began with 3 min of rest and a 3 min ‘unloaded’ warm up, then participants performed the ramp section of the test to exhaustion. The workload during the ramp increased by between 5 and 15 watts per minute, depending on the fitness status of the participant. V̇O_2_peak was defined as the average of the highest exertional oxygen uptake achieved over the last 20 s of exercise. The AT was determined using the modified V-slope method [39], confirmed by patterns of change in ventilatory equivalent and end-tidal gas measurements [40]. In addition to expired air gas analysis, continuous heart rate and peripheral oxygen saturation measurements were made, blood pressure was taken every 3 min, and a 12-lead ECG was continuously monitored.

### Maximum voluntary contraction assessment

During knee extension strength assessment, participants were seated in a supine position on an isokinetic dynamometer (Biodex System 4 Pro, Biodex Medical, Shirley, NY, USA). The patient’s right leg was then securely attached via strapping to the dynamometer knee extension lever arm, whilst ensuring the axis of rotation of the knee joint aligned with the rotational axis of the dynamometer. Inextensible straps were fixed across the hip, distal thigh and chest to reduce extraneous synergistic movements undertaken during maximal contraction. Following the initial setup, participants were briefed on the MVC protocol, which was then followed by a series of warm-up knee extension isometric contractions set at 80°. Each contraction lasted two to three seconds in duration and built up towards to a self‐perceived 50% maximal exertion, ensuring the participant was warmed up prior to maximal exertion. Prior to commencement of the MVC protocol, the investigators confirmed the participants were feeling no discomfort/pain and instructed them to stop exertion if any discomfort/pain was reported during the main protocol. The MVC protocol consisted of two to three isometric knee extension at 80° with 5–10 min rest between contractions. Torque was acquired from the dynamometer and analysed with supplementary software (Biodex Advantage software, Biodex Medical, Shirley, NY, USA). MVCs were repeated if greater than 10% of their previous effort and optimal torque was selected as the highest MVC. Participant’s RTD was calculated using the highest recorded MVC, through utilising the slope of the torque curve from the onset of contraction at an interval of 0–200 ms.

### Assessment of skeletal muscle size and quality

Measurement of muscle size was ascertained through the utilisation of validated methodologies of both VL muscle volume and fascicle length (Lf) in order to calculate the PCSA [41] of the VL, (Muscle Volume ÷ Lf), which is linearly associated with isometric strength [42]. PCSA was then subsequently used alongside knee extension MVC assessment to calculate muscle quality (MVC ÷ VL PCSA).

VL muscle volume was estimated from a single anatomical cross sectional area (ACSA) slice at 50% of muscle length using B-mode ultrasonography (MyLab Gamma, Esaote Biomedica, Genoa, Italy) [43]. Participants lay supine with their knee fully extended for ~ 20 min to avoid fluid shifts [44, 45]. B-mode ultrasonography was then used to ascertain both the proximal insertion (0% of total length) and distal insertion (100% of total length) of the VL on their right leg, where the location of 50% of VL muscle length (*L*) and a line between medial to lateral border (ultrasound probe path) of the VL were marked upon the participants’ leg. VL ACSA was measured using software for panoramic reconstruction of images (VPAN) [46, 47]. The ultrasound probe (7.5 MHz linear array probe, 38 mm wide), was held perpendicular to the muscle and moved with a constant speed and light pressure to avoid compression along the predefined ultrasound path from the lateral to the medial border of the muscle. Analysis of the VL ACSA was conducted offline using the analysis software IMAGEJ (1.45 s; National Institutes of Health, Bethesda, MD, USA). All scans were performed and analysed by the same researcher.

Skeletal muscle Lf of the VL was measured at rest using B-mode ultrasonography with the probe positioned at 50% of the VL length, at mid muscle belly in the sagittal plane. Images were extrapolated from the capturing software and analysed offline. Three clearly visible fascicles with at least 60% of the chosen fascicle visible within the scanning window, defined from the deep to the superficial aponeurosis, were analysed and the mean values of Lf were recorded. Linear extrapolation was undertaken on fascicles that extended beyond the edge of the screen, in line with previous methodology [43].

### Physical activity monitoring

Following completion of the exercise testing protocol, participants were fitted on the anterior thigh (50% of greater trochanter to femoral condyle distance) with a tri-axial GeneActiv Original accelerometer (Activinsights Ltd., Kimbolton, UK) using two waterproof adhesive patches (Tegaderm Film, 3 M, North Ryde, Australia) in line with previous accelerometry physical studies [48]. The accelerometer frequency was recorded at 60 Hz and was worn for between six and seven consecutive days. On return of the accelerometer, the data was downloaded and converted to 60-s epoch files (GENEActiv software version 3.3, Activinsights Ltd., Kimbolton, United Kingdom). Analysis of the data was conducted using GENEActiv macro file version 9, using validated activity cut-off points [49].

### Quality of life and pain assessment

Prior to exercise testing, Health-Related Quality of Life was estimated using the 36-Item Short Form Health Survey questionnaire (SF-36) [50] and pain was assessed using the numeric pain rating scale [51]. The day after testing, patients were contacted via telephone to assess if they had any adverse reactions to the exercise trials, including further assessments of pain using the numeric pain rating scale. If an adverse reaction was reported the patient was contacted on subsequent days until symptoms subsided.

### Data and statistical analysis

Predicted CPET values were calculated using published normative data; peak work rate [52], V̇O_2_peak [53], maximum voluntary ventilation (MVV) [54], maximum heart rate (HR) (220-age) [55], and strength [25].

SPSS (v26, IBM) was used for data analysis, with the significance set at *p* < 0.05. Data was checked with a Shapiro–Wilk test (sample n < 50) for normality of distribution. If parametric assumptions were accepted, paired samples t-tests assessed whether measured values differed from results calculated using prediction equations, Pearson’s correlations were used to test the strength of associations, and data are presented as mean (standard deviation). If parametric assumptions were breached, a paired samples Wilcoxon test was used to assess differences, Spearman’s rank correlation was utilised, and data are presented as median (IQ range).

## Supplementary Information

**Additional file 1: Table S1.** Self-reported health-related quality of life, ordered by V̇O2peak (from highest to lowest).

## Data Availability

The datasets used and/or analysed during the current study are available from the corresponding author on reasonable request.
