# Supplementary material for: Aerobic capacity and skeletal muscle characteristics in glycogen storage disease IIIa: an observational study
Source: Orphanet J Rare Dis. 2022 Jan 31;17:28. doi: 10.1186/s13023-022-02184-1 (PMC8802498; doi:10.1186/s13023-022-02184-1)
Supplement: Supplementary file 1 — Additional file 1: Table S1. Self-reported health-related quality of life, ordered by V̇O2peak (from highest to lowest). [file 13023_2022_2184_MOESM1_ESM.docx]

**Supplementary table 1: Self-reported health-related quality of life, ordered by V̇O_2_peak (from highest to lowest)**

| **Participant** | **Physical functioning** | **Role limitations due to:** | | **Energy/fatigue** | **Emotional well-being** | **Social functioning** | **Pain** | **General health** |
| --- | --- | --- | --- | --- | --- | --- | --- | --- |
|  |  | **physical health** | **emotional problems** |  |  |  |  |  |
|  |  |  |  |  |  |  |  |  |
| **1** | 70 | 75 | 100 | 35 | 92 | 100 | 77.5 | 50 |
| **2** | 70 | 0 | 100 | 45 | 48 | 75 | 22.5 | 45 |
| **3** | 90 | 0 | 0 | - | - | - | 45 | 30 |
| **4** | 10 | 0 | 100 | 25 | 68 | 50 | 90 | 35 |
| **5** | 0 | 0 | 33 | 5 | 56 | 12.5 | 10 | 10 |
| **6** | 35 | 50 | 100 | 60 | 88 | 62.5 | 90 | 35 |
| **7** | 10 | 0 | 0 | 5 | 40 | 12.5 | 32.5 | 10 |
|  |  |  |  |  |  |  |  |  |
| **Mean (SD)** | 41 (36) |  |  | 29 (22) | 65 (21) | 52 (35) | 53 (33) | 31 (16) |
| **Median (IQ range)** |  | 0 (50) | 100 (100) |  |  |  |  |  |
